# Preparation of Sepiolite Nanofibers Supported Zero Valent Iron Composite Material for Catalytic Removal of Tetracycline in Aqueous Solution

**DOI:** 10.3389/fchem.2021.736285

**Published:** 2021-09-08

**Authors:** Xiaoyu Han, Hong Zhang, Caihong Zhang, Yan Zhao, Na Zhang, Jinsheng Liang

**Affiliations:** ^1^ Key Laboratory of Special Functional Materials for Ecological Environment and Information (Hebei University of Technology), Ministry of Education, Tianjin, China; ^2^ Institute of Power Source and Ecomaterials Science, Hebei University of Technology, Tianjin, China

**Keywords:** sepiolite, nanoscale zero valent iron, green synthesis, catalytic performance, antibiotic degradation

## Abstract

The heavy use of antibiotics in medicine, stock farming and agriculture production has led to their gradual accumulation in environmental media, which poses a serious threat to ecological environment and human safety. As an efficient and promising catalyst for the degradation of antibiotics, nanoscale zero valent iron (nZVI) has attracted increasing attention in recent years. In this study, sepiolite nanofiber supported zero valent iron (nZVI/SEP) composite was prepared via a facile and environmentally friendly method. The nZVI particles (with size of 20–60 nm) were dispersed evenly on the surface of sepiolite nanofibers, and the catalytic performance for the removal of tetracycline hydrochloride (TC-HCl) in aqueous system was investigated. The effect of nZVI loading amount, catalyst dosage, H_2_O_2_ concentration and pH on the removal efficiency of TC-HCl were studied. It was revealed that the sepiolite supporter effectively inhibited the agglomeration of nZVI particles and increased the contact area between contaminant and the active sites, resulting in the higher catalytic performance than pure nZVI material. The TC-HCl removal efficiency of nZVI/SEP composite was up to 92.67% when TC-HCl concentration of 20 mg/L, catalyst dosage of 1.0 g/L, H_2_O_2_ concentration of 1.0 mM, pH value of 7. Therefore, the nZVI/SEP composites possess high catalytic activity for TC-HCl removal and have great application prospects in antibiotic wastewater treatment.

## Introduction

In recent years, refractory organic pollutants such as antibiotics (Ouyang et al., 2019; Chen et al., 2020a), mycotoxins (Li et al., 2018; Sun et al., 2020) and drugs (Daneshkhah et al., 2017; Phasuphan et al., 2019) have become emerging environmental issues because of the rapid development of pharmaceutical industry, agriculture and animal husbandry. Particularly, the abuse of antibiotics has led to the gradual accumulation in soil and water environments, which enhances the bacterial resistance and endangers various ecosystems (Dong et al., 2018; Pirsaheb et al., 2019). Among various antibiotics, tetracycline is the most extensively used antibiotic around the world because of its low cost and high antimicrobial activities (Xu et al., 2020; Zhao et al., 2020). However, only a small fraction of tetracycline can be metabolized or adsorbed by humans or animals, 50–80% residuals and metabolites enter into the environment. Thus, tetracycline antibiotics with low biodegradability have been frequently detected in soil and water (Hou et al., 2020; Zhang et al., 2021). Tetracycline accumulation in the environment readily leads to cause bacterial resistance to antibiotics, which poses a serious threat to human health and ecological security. Consequently, the removal of antibiotics has been paid close attention by the scientific researchers in the fields of biology, chemistry, medicine and environment. General technologies, such as biodegradation, absorption, coagulation and sedimentation, have a limited impact on the removal of various antibiotics. In comparison, the advanced oxidation processes (AOPs) can generate highly reactive free radicals to efficiently degrade antibiotics (Huang et al., 2020).

Nanoscale zero-valent iron (nZVI) has been proved as an effective material for the removal of organic and inorganic contaminants because of its high reactivity and low toxicity of reaction products (Hemmat et al., 2021). In Fenton-like process, nZVI can be acted as a source of Fe^2+^. The produced Fe^2+^ reacts with hydrogen peroxide (H_2_O_2_) to form OH, which has strong oxidizing ability to efficiently degrades and mineralize organic contaminants in aquatic system (Pirsaheb et al., 2019; Zhou et al., 2019). However, nZVI materials exhibit certain deficiencies in practical applications including strong tendency to aggregation and oxidation, secondary iron pollution, difficult separation and recovery (Bossa et al., 2017; Nasiri et al., 2019; Gopal et al., 2020). Particularly, the aggregation of nZVI particles will significantly affect the mobility and effective surface area to reduce the catalytic activities. To overcome these problems, it has been proposed to load nZVI particles on various supports like clay minerals (Frost et al., 2010; Bao et al., 2019), zeolite (Suazo-Hernandez et al., 2019), silica materials (Guo et al., 2021), activated carbon (Huang et al., 2019) and biochar (Zhang et al., 2020).

Sepiolite is a natural hydrated magnesium silicate mineral with chemical formula of Mg_8_Si_12_O_30_(OH)_4_(OH_2_)_4_ 8H_2_O. And it is a 2:1 phyllosilicate composed of two Si-O tetrahedral layers and an intermediate layer of Mg-O(OH) octahedral (Rao et al., 2018). The sepiolite with abundant micropores and channels possesses high surface area and strong absorbability, which should be in favor of conducting contaminants to the reactive cites of the composite materials resulting in the improvement of degradation efficiency (Ezzatahmadi et al., 2017). Daneshkhah et al. (2017) synthesized sepiolite-supported nanoscale zerovalent iron by sodium borohydride reduction method to removal metoprolol from water. Habish et al. (2017) studied the composites with different radio of sepiolite and nanoscale zerovalent iron to achieve the best nZVI dispersibility and the highest adsorption capacity for Cd^2+^. However, there are few studies on the utilization of nZVI-sepiolite composites for the removal of antibiotic contaminants.

Because no toxic reducing agents (like NaBH_4_) or nitrogen protection and other harsh processing conditions are needed, green synthesis is more suitable for large-scale production of nZVI materials (Badmus et al., 2018; Mondal et al., 2020). Martins et al. (2017) compared the environmental impacts and costs between sodium borohydride reduction (traditional synthesis) and green synthesis method by life cycle assessment (LCA). The results showed that the green synthesis presented 50% lower environmental impacts than the sodium borohydride reduction, and the traditional synthesis was much more expensive than the green synthesis (roughly eight times higher). In this study, the sepiolite nanofiber supported nZVI composites (nZVI/SEP) were synthesized by green method. In the course of green synthesis, the biologically-active substances from plant extracts (green tea, coffee, eucalyptus leaves, grape leaves, pomegranate leaves, etc.) can both reduce the Fe^2+^ or Fe^3+^ and effectively prevent nanoparticles from oxidation. Among them, green tea extracts are kinds of excellent reducing and capping agents owing to the large amounts of polyphenols substances. The prepared nZVI/SEP composites were employed as Fenton-like catalyst to remove tetracycline hydrochloride (TC-HCl) from aqueous solutions. The surface morphology and micropore structure of the nZVI/SEP composite were characterized. And the effects of reaction time, TC-HCl initial concentration, catalyst dosage, H_2_O_2_ concentration, and pH on TC-HCl degradation process were investigated.

## Materials and Methods

### Materials

The natural sepiolite used in the experiments was obtained from Nanyang of Henan Province. Its chemical composition (wt%) was: SiO_2_, 39.20; MgO, 17.00; CaO, 32.40; Al_2_O_3_, 6.20; Fe_2_O_3_, 3.46, MnO, 0.40; TiO_2_, 0.27; K_2_O, 0.21 and Na_2_O, 0.20. The natural sepiolite was firstly treated with 2 mol/L HCl solution in a magnetic stirrer for stirring 12 h at room temperature for impurity removal and activation. The solid was filtered and centrifuged, washed several times with deionized water, and then dried at 80°C for 10 h. All chemical regents used in this work, tetracycline hydrochloride (C_22_H_24_N_2_O_8_ HCl, 98% purity), H_2_O_2_ (30 wt%), HCl (37 wt%), and FeCl_3_ 6H_2_O were of analytical grade and used without further purification.

### Preparation of nZVI/SEP Composite

To prepare green tea extract, dried green tea (15 g) was added to 250 ml deionized water in round bottom flask. The mixture was then heated up to boiling at 80°C for 1 h and then filtering the extract to remove tea leaves. The green tea extracts were stored at 4°C until further use.

For the synthesis of nZVI/SEP composite, a certain amount of sepiolite was added to the conical flask and mixed with 0.1 M FeCl_3_ solution (the SEP:nZVI mass ratio was 10:1, 8:1, 5:1, 2:1, respectively), and the equal volume of green tea extract was dropped into the flask while continuously stirring at room temperature and the solution immediately turned black. The black particles were collected by centrifuging followed by washing several times with deionized water and absolute ethanol. Finally, the obtained particles were dried under vacuum at 60°C for 12 h. The obtained composites were denoted as 0.1 nZVI/SEP, 0.125nZVI/SEP, 0.2nZVI/SEP, 0.5 nZVI/SEP, respectively.

### Characterization of nZVI/SEP Composite

The X-ray diffraction (XRD) patterns were performed using a Bruker D8 Advance diffractometer with Cu Kα radiation over 2θ ranging from 10° to 90°. The morphologies and structures of the samples were observed by scanning electron microscopy (SEM, FEI Nano SEM450) and transmission electron microscopy (TEM, Philips Tecnai G2 F20). The X-ray photoelectron spectroscopy (XPS, Thermo Fisher ESCALAB 250Xi) was used to determine the elemental composition of sample surface. Fourier transform infrared spectroscopy (FTIR) spectra were recorded using a Bruker V80 spectrometer in the range of 4,000–400 cm^−1^. Nitrogen adsorption desorption isotherms were measured at −196°C using a Quantach rome Autosorb iQ2 analyzer. The specifc surface area of the samples was calculated according to the Brunauer-Emmett-Teller (BET) method, and the pore volumes were taken at P/P0 = 0.990 single point. The pore size distributions were calculated by the Barrett-Joyner-Halenda (BJH) method.

### Batch Experiments

TC-HCl was chosen as the target pollutant to evaluate the catalytic performance of nZVI/SEP. For each experiment, 100 ml of TC-HCl solution with a certain concentration (10, 20, 30, 50 and 80 mg/L) was added to the conical flasks at room temperature. After that, certain dosages of catalyst (0.5, 1.0, and 1.5 g/L) and H_2_O_2_ (0.5, 1.0 and 1.5 mM) were added to the solution. The solution pH (4, 5 and 6) was adjusted by 0.1 M HCl and 0.1 M NaOH solution. After the obtained mixture was shaken for a predetermined time (30, 60, 90, 120, 180 and 240 min), the supernatant solution was separated from the catalyst by centrifugation at 5,000 rpm for 5 min. Then the concentration of TC-HCl supernatant was measured by UV–vis spectrophotometer at the 357 nm wavelength. The removal efficiency (R) of TC-HCl was calculated as follows:

$$R\left(\%\right)=\frac{C_{0}-C_{t}}{C_{0}}\times 100\%$$ (1)

where C_0_ (mg/L) is the initial concentration and C_t_ (mg/L) is the concentration of TC-HCl at reaction time t (min).

## Results and Discussion

### Characterization of nZVI/SEP Composite

**FIGURE 1:**
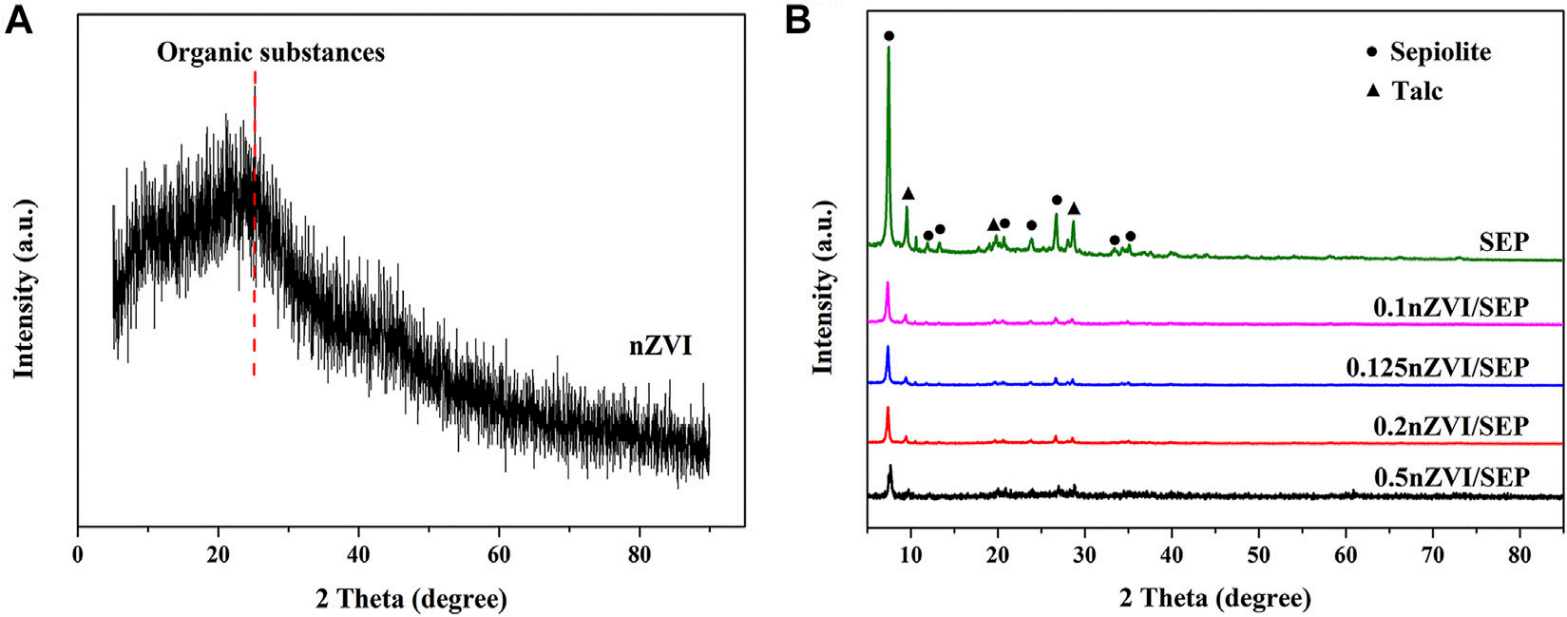
XRD patterns of **(A)** nZVI, **(B)** SEP and nZVI/SEP composites.

Figure 1 shows the XRD pattern of the sepiolite, synthesized nZVI and nZVI/SEP composite. The XRD pattern shows the characteristic peak of sepiolite at 2θ = 7.3°, which is consistent with the reference material. A little amount of talc impurity is also observed in the SEP sample. There is no sharp diffraction peak observed in the pattern of nZVI, indicating that the synthesized nZVI is essentially amorphous. And the broad shoulder peak at around 2θ = 22.6° can be identified as amorphous carbon, which suggests that organic molecules from the green tea extract have successfully combined with nZVI and coated the surface of the nZVI particles. Besides, typical Fe^0^ diffraction peaks cannot be observed in the patterns of nZVI/SEP composites. It is probably because the low loading amount and absence of crystallinity of iron. Similar results are reported in literatures (Liu et al., 2018; Yazdani et al., 2019; Lin et al., 2020).

**FIGURE 2:**
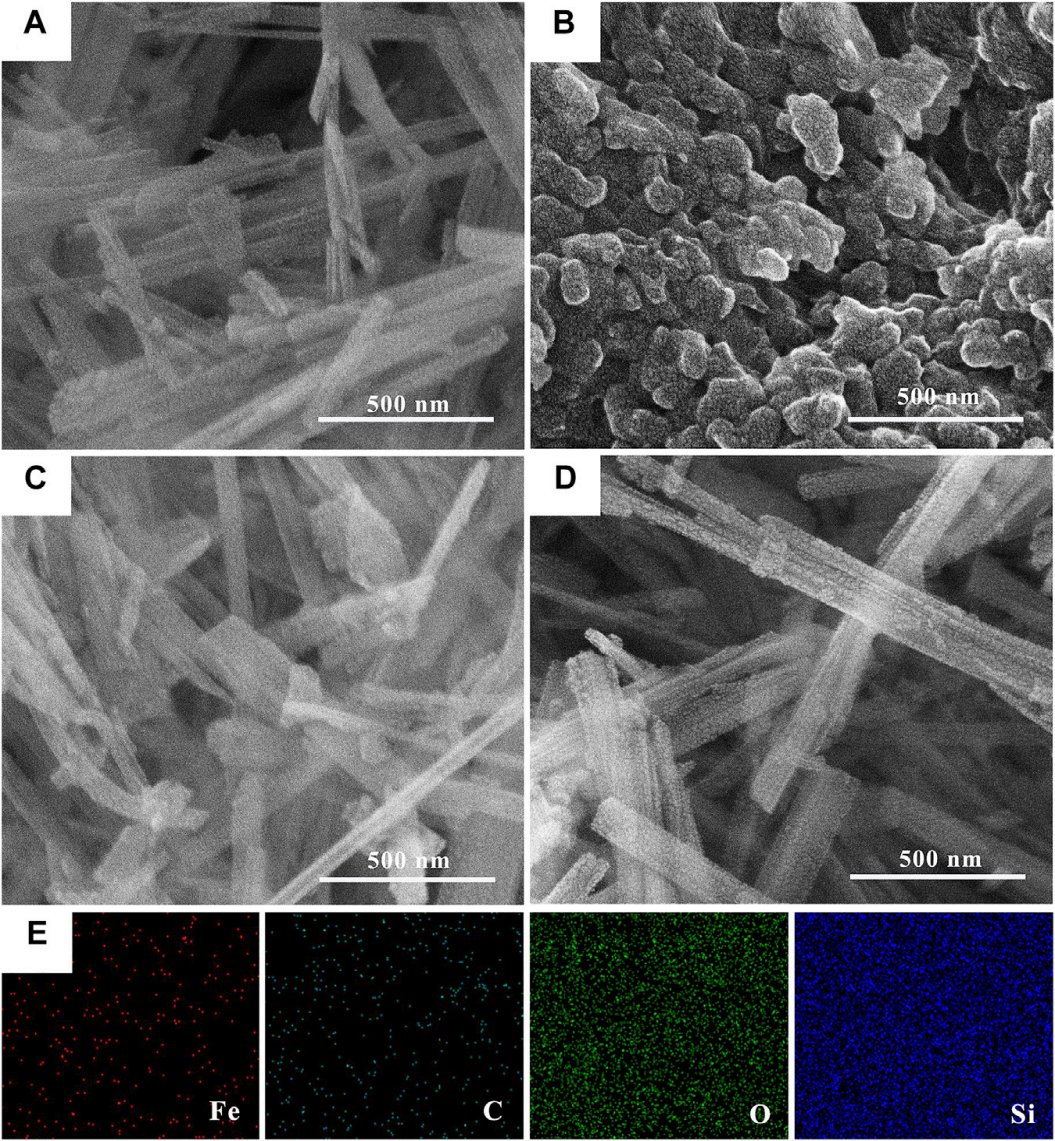
SEM images of **(A)** SEP, **(B)** nZVI, **(C, D)** nZVI/SEP composites and **(E)** the element mapping of nZVI/SEP composites.

The surface morphologies of SEP, nZVI and nZVI/SEP composite are presented in Figure 2. The SEM images reveal that the sepiolite particles exhibit a characteristic fibrous morphology, and the nZVI particles shows obviously aggregated spherical particles, with sizes between 100 and 200 nm. As for the nZVI/SEP composite, the surface of sepiolite observed with conspicuous granule, demonstrating that the nZVI particles are uniformly distributed throughout the sepiolite fibers without aggregation. And the size of nZVI particles in nZVI/SEP composites is less than 100 nm, which are significant decreased compared with the pure nZVI. The elemental mapping image of nZVI/SEP further reveals the homogeneous immobilization of nZVI particles on nZVI/SEP composite surface. From the distribution of elements Fe and C, it can be concluded that the oxidized polyphenols from green tea act as stabilizing and capping agent coated on the surface of nZVI particles.

**FIGURE 3:**
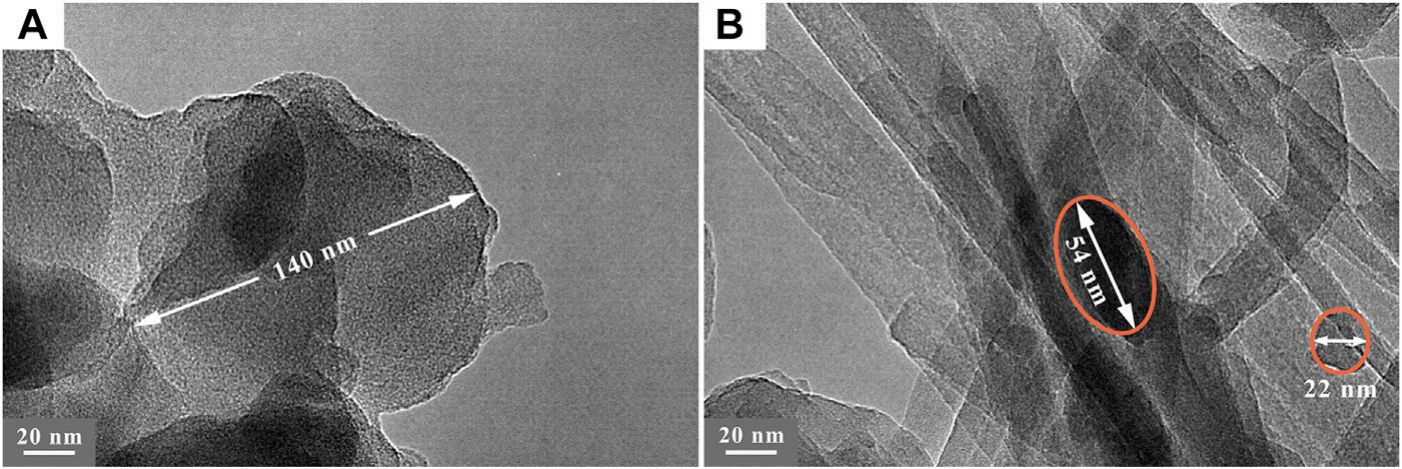
TEM images of **(A)** nZVI and **(B)** nZVI/SEP composite.

The particle shape and size of nZVI, dispersity of nZVI/SEP composite are further investigated by TEM. From Figure 3A, the nZVI particles are approximately spherical in shape with the diameter around 140 nm, and the Fe^0^ grains are encapsulate by a layer of membranous substance. The membranous substance is considered to amorphous carbon derived from green tea extract, which is consistent with the results of element mapping. Although polyphenols in green tea as stabilizing agent can reduce electrostatic repulsion and steric hindrance, some aggregation still can be observed in nZVI sample because of the magnetic interaction of nZVI particles. In Figure 3B, the nZVI particles are well dispersed and attached to sepiolite fibers as individual spherical shaped particles, and the particle size is much smaller than the pure nZVI particles. It indicates that sepiolite fiber supported nZVI described in this work is an effective approach to improve the dispersion properties and applied performances of Fe^0^ nanoparticles.

**FIGURE 4:**
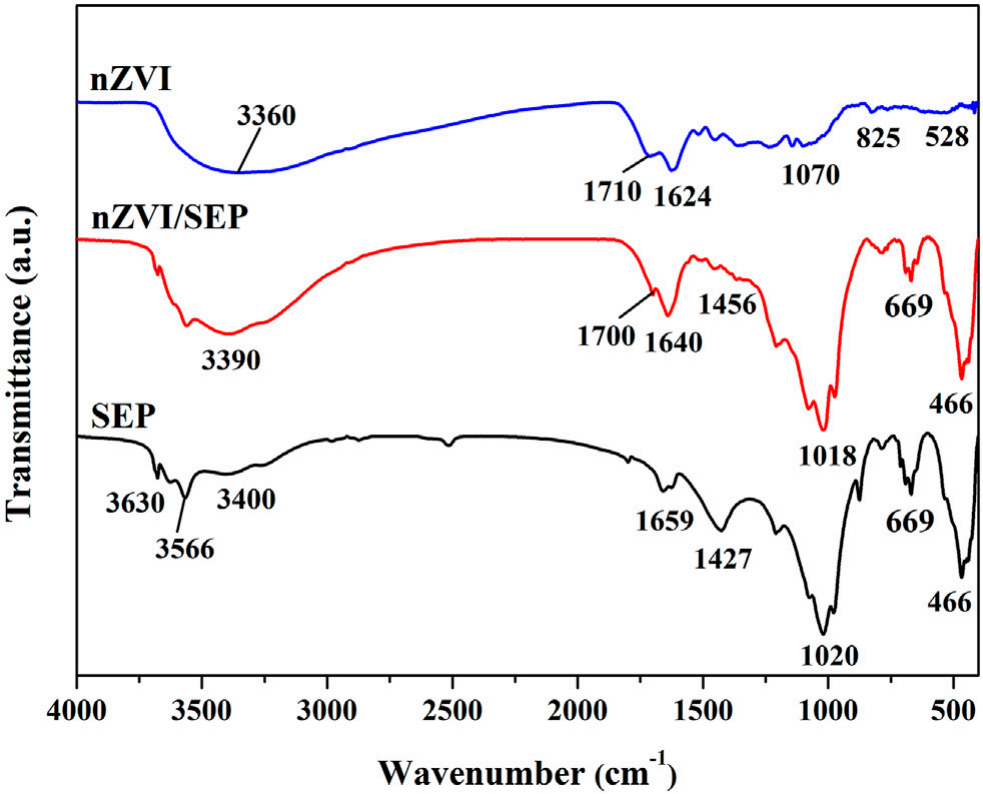
FTIR spectra of SEP, nZVI and nZVI/SEP samples.

The FTIR spectra of SEP, nZVI and nZVI/SEP are carried out in the range of 400–4,000 cm^−1^ and the results are shown in Figure 4. The peaks in the region around 3,400–3,600 cm^−1^ corresponds to O-H stretching vibration owing to the water molecule and hydroxyl-band (Mg_3_OH) of SEP, whereas the peaks at 1,659 and 669 cm^−1^ correspond to O-H bending vibration (Daneshkhah et al., 2017; Wu et al., 2017). The peak at 1,020 cm^−1^ is assigned to the Si-O stretching vibration of sepiolite tetrahedral sheets. And the peak at 466 cm^−1^ is assigned to the Si-O-Si bending vibration (Thao et al., 2018). The peak at 1,427 cm-1 relates to the C-O stretching vibration of carbonate impurity (Ma et al., 2017). For nZVI sample, the broad peak around 3,360 cm^−1^ is attributed to the O-H stretching vibration of polyphenols from green tea extract in synthesis of Fe nanoparticles. The peak at 1710 cm^−1^ is assigned to C=O stretching vibration of carbonyl groups derived from polyphenols. Moreover, the peak at 1,624 cm^−1^ can be ascribed to the C=C stretching vibration of aromatic ring (Wang et al., 2017), while the peak at 1,070 cm^−1^ is ascribed to C-O-C stretching vibration (Liu et al., 2017). In addition, the weak absorption band at 825 and 528 cm^−1^ is attributed to Fe-O stretching vibration of Fe oxide (Wang et al., 2020), which confirm properly the synthesis of nZVI particles. After nZVI loaded on the surface of sepiolite, it is observed that several peaks are disappeared or shifted because of the interaction of the functional groups existing on SEP and nZVI. These changes prove the successful loading and immobilization of nZVI particles on sepiolite fibers.

**FIGURE 5:**
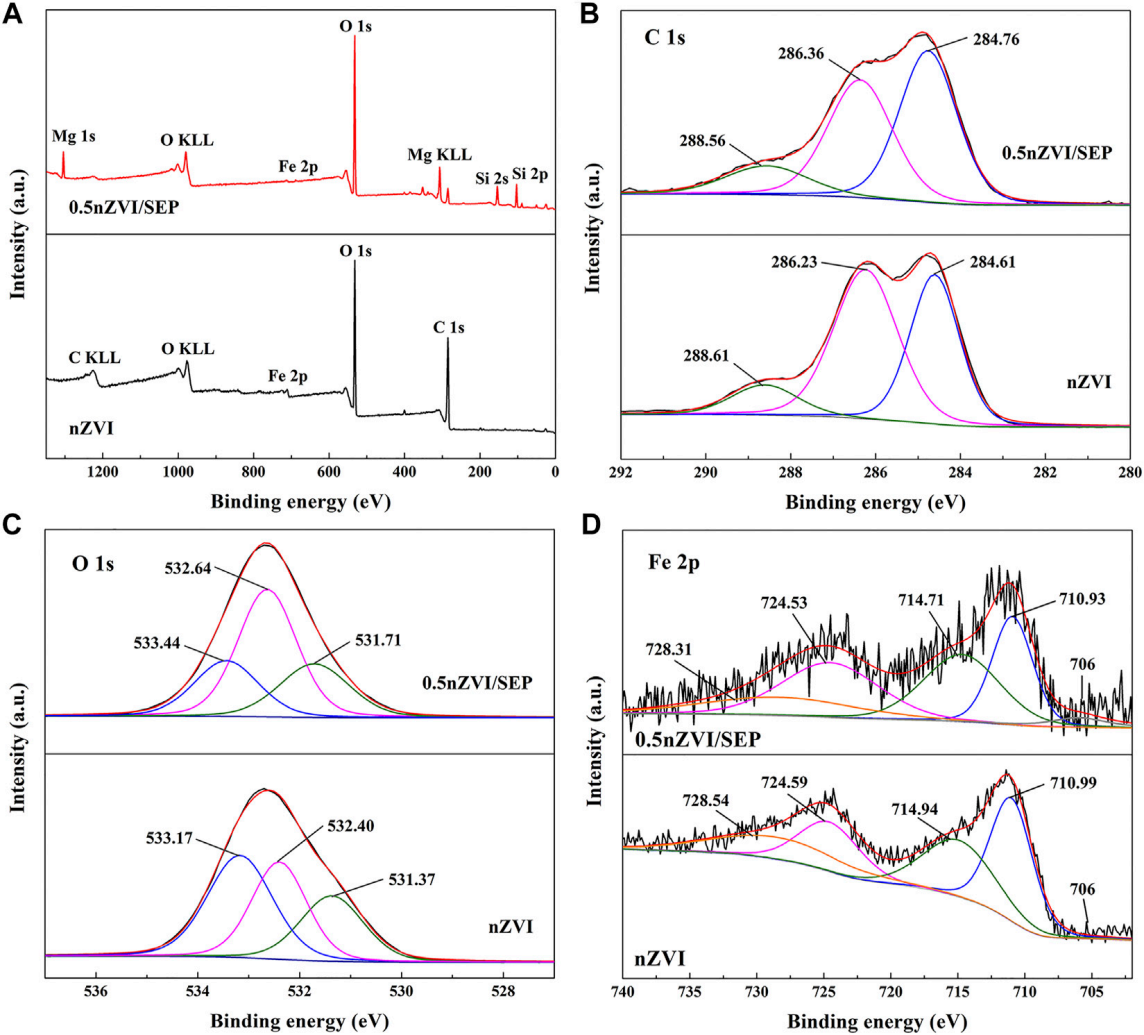
XPS spectra of **(A)** full-range scan spectra of nZVI and nZVI/SEP, and **(B)** Fe 2p, **(C)** C 1s and **(D)** O 1s of nZVI/SEP composite.

XPS is performed to investigate the composition and chemical states of nZVI and nZVI/SEP as shown in Figure 5. From the full scan spectra (Figure 5A), it can be clearly observed that synthesized nZVI is composed of Fe, C and O, while nZVI/SEP composite is composed of Fe, Si, Mg, O and C. The result indicates that Fe^0^ is successfully synthesized *via* green method and loaded on the surface of sepiolite. Figure 5B presents the C 1s spectra, and the binding energy of 284.61, 286.23, and 288.61 eV are contributed to C-C, C-O and C=O, respectively (Balachandramohan and Sivasankar, 2018). As shown in Figure 5C, the O 1s spectra at 533.17 eV indicates the oxygen bonding to carbon. The peak at 531.71 eV is assigned to the lattice oxygen (O^2-^) of metal oxide, and the peak at 532.40 eV is owing to the hydroxyl groups (-OH) (Liu et al., 2018). Consequently, the spectra of C 1s and O 1s confirm that some biomolecules (mainly amorphous carbon) from green tea extract are capped on the surface of nZVI particles, the result is consistent with TEM analysis. For the Fe 2p high-resolution spectrum of nZVI (Figure 5D), two peaks at 710.99 eV (Fe 2P_3/2_) and 724.59 eV (Fe 2p_1/2_) are assigned to Fe_2_O_3_. And the binding energy at 714.94 eV (Fe 2P_3/2_) and 728.54 qeV (Fe 2p_1/2_) are attributed to octahedrally-coordinated Fe^3+^ from hydroxides (FeOOH) (Chen et al., 2020a). Moreover, the weak peaks around 706 eV in nZVI and 0.5nZVI/SEP are also observed, which correspond to the Fe 2P_3/2_ for Fe^0^. Because of the high reactivity of nZVI particles, the Fe^0^ is easily oxidized in the process of preparation and storage, thus forming a layer of iron oxides on the surface of Fe^0^. The presence of surface substances (amorphous carbon and iron oxides) of nZVI particles limit the Fe^0^ detection, since XPS is a sensitive surface analysis technique with only 2–5 nm detection depth (Ma et al., 2016; Nguyen et al., 2019).

**FIGURE 6:**
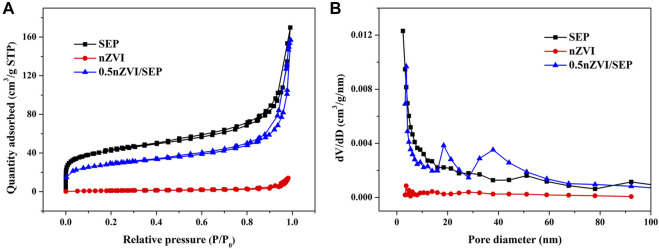
**(A)** N_2_ adsorption-desorption isotherms and **(B)** pore size distribution of SEP, nZVI and nZVI/SEP composite.

Catalytic activity of heterogeneous catalysts is closely related to their pore structure, which supply active sites for contaminants. Thus, N_2_ adsorption-desorption analyses of SEP, nZVI and nZVI/SEP composite were carried out, and the isotherms as well as the pore size distribution are shown in Figure 6. According to the IUPAC classification, the isotherms of all three samples belong to the typical type Ⅳ and H3 hysteresis loops, indicating the presence of mesoporous structure. However, the adsorption amount of pure nZVI is well below than SEP and nZVI/SEP composite, this is probably because nZVI particles are tend to agglomerate, resulting in the lower specific surface area. And the pore size distribution diagram indicates that the pore diameter of these samples is mainly distributed between 3 and 50 nm, which further proves that the nZVI/SEP composite have abundant mesopores and wide pore size distribution.

Table 1 shows the BET surface area, total pore volume and average pore size of SEP, nZVI and nZVI/SEP composites. The BET results reveal that the specific surface area of nZVI/SEP composite decreases with the increase of loading amount of nZVI, which may be attributed to the partial blocking of the pores by nZVI loaded on sepiolite surface. Besides, the pore size of nZVI/SEP composites increases gradually due to the formation of larger mesopores accumulated by nZVI on sepiolite fibers. Compare with the pure nZVI, the nZVI/SEP composites exhibit higher specific surface area and larger number of mesopores, which confirming that nZVI particles are well dispersed on sepiolite surface without significant aggregation. Therefore, porous structure of nZVI/SEP composites provide a large number of reaction sites for better contract between the catalyst and pollutants.

**TABLE 1:** Pore structural characteristics of SEP, nZVI and nZVI/SEP composites.

Sample	S_BET_ (m^2^/g)	V (cm^3^/g)	D_BJH_ (nm)
SEP	152.78	0.2581	6.7566
nZVI	4.04	0.0216	21.3890
0.1n ZVI/SEP	122.90	0.2456	7.9919
0.125 nZVI/SEP	115.36	0.2397	8.2650
0.2 nZVI/SEP	104.55	0.2341	8.7187
0.5 nZVI/SEP	101.35	0.2285	9.0194

### TC-HCl Removal by Synthesized Catalyst

Tetracycline hydrochloride is amphoteric compound with multiple ionizable functional groups. When pH is below 3.30, TC-HCl primary exists in the form of cation, zwitterion as main form when pH value is between 3.30 and 7.68, while forming the anion when pH is above 7.68 (Yang et al., 2018; Sun et al., 2021). In this study, the removal efficiency of TC-HCl was investigated under various systems, catalyst dosage, H_2_O_2_ concentration and pH value.

**FIGURE 7:**
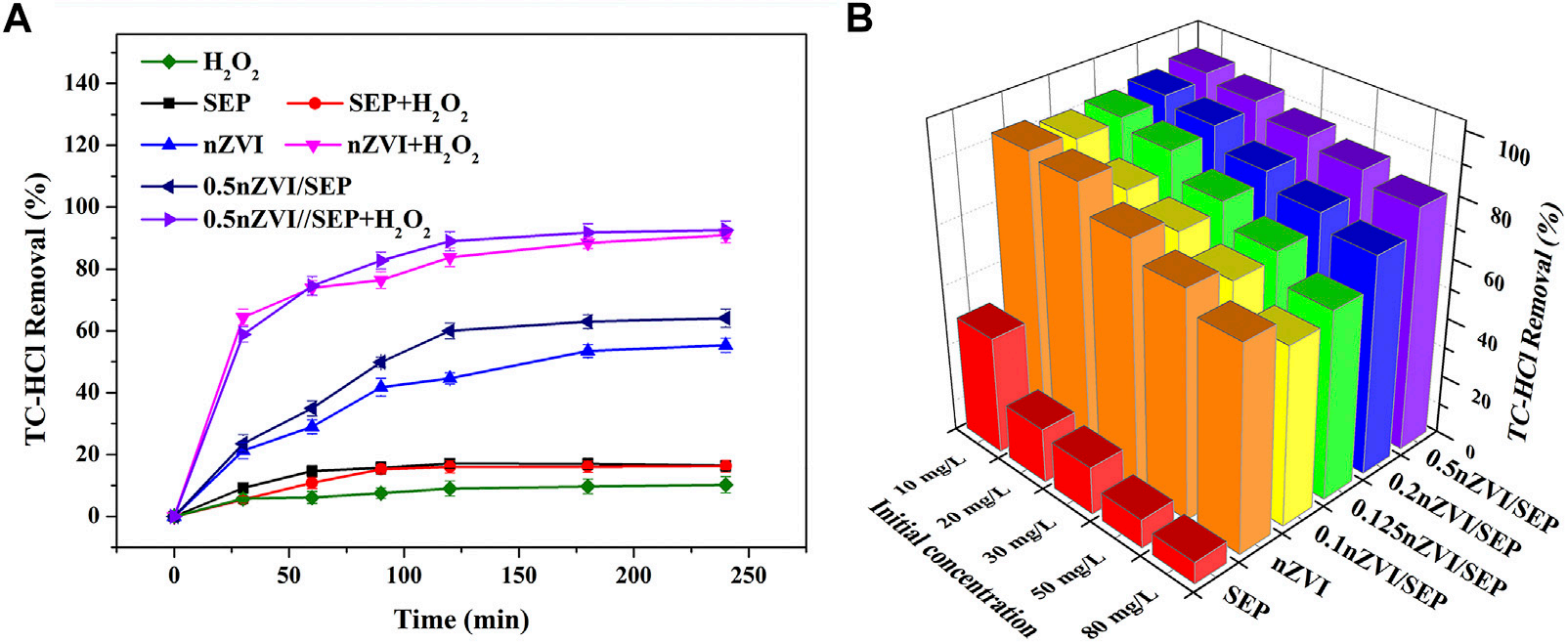
**(A)** TC-HCl removal in different systems, **(B)** TC-HCl removal efficiency by different catalysts. (Experimental conditions: TC-HCl concentration = 20 mg/L, catalyst dosage = 0.1 g/L, H_2_O_2_ concentration = 1 mM, pH = 7, room temperature).

The effect of different systems on the removal of TC-HCl are shown in Figure 7A. There is no obvious removal efficiency for TC-HCl of H_2_O_2_, suggesting that H_2_O_2_ alone is unable to degrade TC-HCl because of its poor oxidation ability. And SEP shows poor TC-HCl adsorption, only 16.48% of TC-HCl is removed within 240 min. Instead, nZVI and nZVI/SEP composite show good degradation effect for TC-HCl, the final removal efficiency of 240 min is about 55.37 and 64.15%, respectively. Furthermore, TC-HCl removal efficiency is dramatically improved after adding H_2_O_2_ into the nZVI and nZVI/SEP system, and the removal efficiency can be up to 90.93 and 92.67%, respectively. Considering the minor role of TC-HCl adsorption, TC-HCl removal is mainly attributed to heterogeneous catalysis, and the presence of zero valent iron is favorable to the Fenton-like reaction. Figure 7B shows the removal efficiency of different catalysts for TC-HCl with different initial concentration of TC-HCl. It is seen that sepiolite support zero valent iron exhibits excellent catalytic performance compared with pure nZVI and SEP. And the TC-HCl removal efficiency of the nZVI/SEP composites significantly improve with the increasing of nZVI loading amounts. When the loading amount of nZVI is 0.2, the removal efficiency of nZVI/SEP composite for TC-HCl with different concentration is higher than pure nZVI. 0.5 nZVI/SEP shows the best degradation effect for different concentrations of TC-HCl. The removal efficiencies of 10 mg/L, 20 mg/L, 30 mg/L, 50 mg/L and 80 mg/L TC-HCl are reach 95.36, 92.67, 88.26, 85.08 and 80.97% respectively. Although the adsorption of TC-HCl on sepiolite is negligible under the condition of different concentrations of TC-HCl, the large specific surface area of sepiolite enhances the initial contact between nZVI particles and TC-HCl, as well as improves the dispersion of nZVI particles, thus increasing the number of active sites as a result.

**FIGURE 8:**
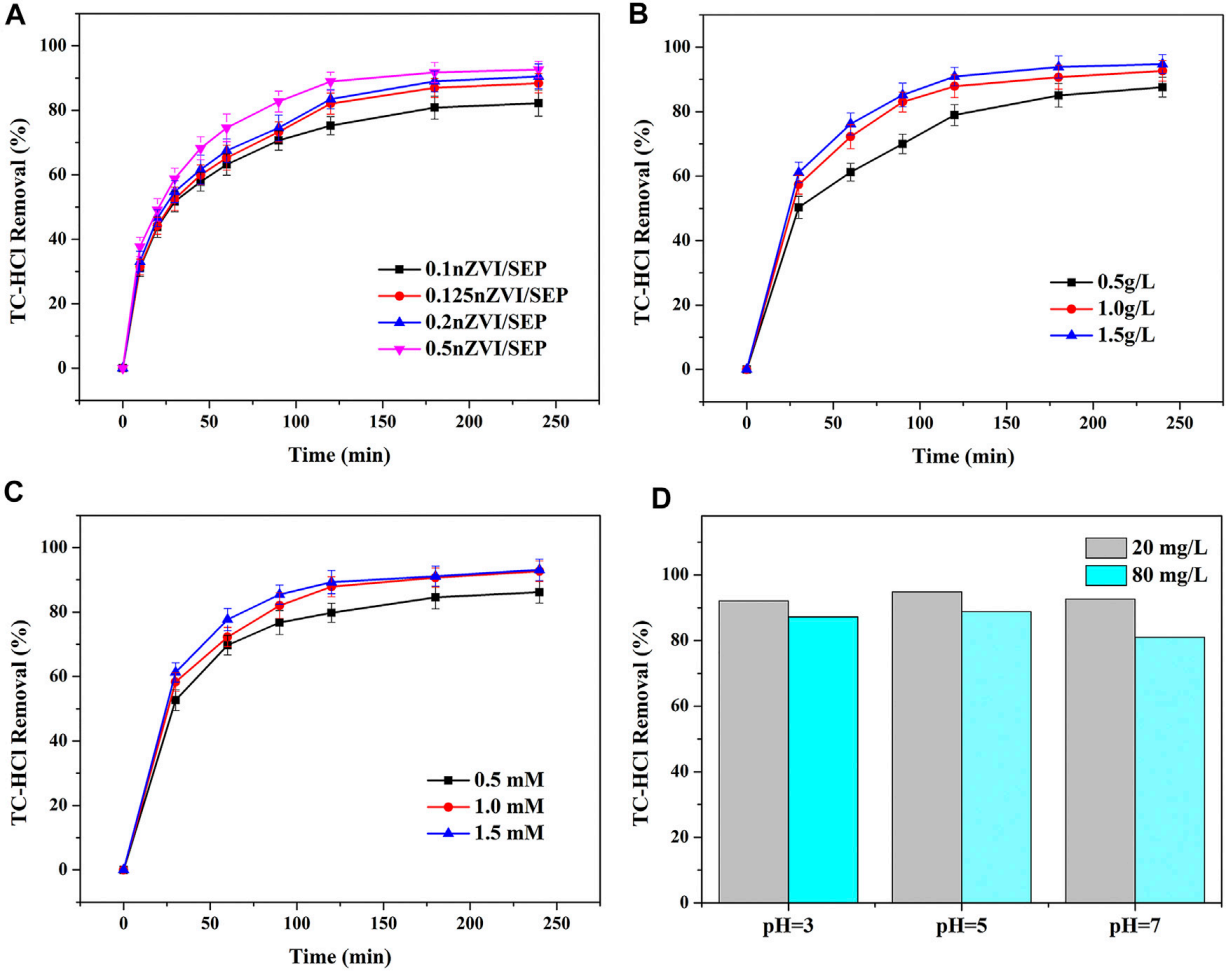
Effect of **(A)** loading amount of nZVI, **(B)** catalyst dosage, **(C)** H_2_O_2_ concentration, **(D)** pH value on TC-HCl removal by 0.5 nZVI/SEP. (Experimental conditions: TC-HCl concentration = 20 mg/L, catalyst dosage = 0.1 g/L, H_2_O_2_ concentration = 1 mM, pH = 7, room temperature).

The relationship between reaction time and removal efficiency of TC-HCl by different nZVI/SEP composites are described in Figure 8A. For four nZVI/SEP composites, the TC-HCl removal curves look similar, and their reaction rates are faster within 60 min and then the removal efficiency gradually increase with the increase of reaction time. The TC-HCl removal efficiency on these samples all can reach over 80% in about 180 min. The catalyst with high loading amount of nZVI have higher degradation efficiency of TC-HCl. However, when the loading amount of nZVI is more than 50%, the removal efficiency for TC-HCl of nZVI/SEP composite may decrease attributed to the aggregation of nZVI at higher content. Therefore, the catalyst of 0.5 nZVI/SEP is selected to study the effects of catalyst dosage, H_2_O_2_ concentration and pH on TC-HCl removal.

The effect of catalyst dosages on TC-HCl removal efficiency is shown in Figure 8B. It can be observed that the TC-HCl removal efficiency increases from 87.62 to 94.77% with the catalyst dosage increasing from 0.5 to 1.5 g/L. It can be explained that the catalytic activity is closely related to the quantity of active sites, higher dosage of catalyst lead to more Fe^2+^ ions generated from nZVI (Eq. 2). Subsequently, Fe^2+^ combines with H_2_O_2_ to create more hydroxyl radicals (Eq. 3) which possess high oxidizing ability toward TC-HCl.

$${\text{Fe}}^{0}{\;+\text{ H}}_{2}{\text{O}}_{2}{\;+\;2\text{H}}^{+}\;\to {\text{ Fe}}^{2+}{\;+\;2\text{H}}_{2}\text{O}$$ (2)

$${\text{Fe}}^{2+}{\;+\text{ H}}_{2}{\text{O}}_{2}\;\to {\text{ Fe}}^{3+}{\;+\cdot \text{OH }+\text{ OH}}^{-}$$ (3)

In the heterogeneous Fenton system, hydroxyl radical is mainly produced from H_2_O_2_, which has a direct influence on the degradation of TC-HCl (Qin et al., 2020; Wu et al., 2020). From Figure 8C, the TC-HCl removal efficiency increases from 86.15 to 92.67% with increasing H_2_O_2_ concentration from 0.5 to 1.0 mM. When the concentration of H_2_O_2_ is low, the degradation of TC-HCl is not complete, which may be due to the lack of enough OH in aqueous solution. With the H_2_O_2_ concentration increasing, the amount of OH in the system also increases, which accelerates the degradation of TC-HCl and improves the removal efficiency of TC-HCl. However, a slight improvement in TC-HCl removal efficiency is observed as the H_2_O_2_ concentration continues rising to 1.5 mM. It is because a limited number of active sites only react with a certain number of H_2_O_2_ molecules, and excessive H_2_O_2_ could not decompose to produce more hydroxyl radicals. Moreover, excessive H_2_O_2_ has been proved to promote the reaction of OH with H_2_O_2_ and HO_2_, resulting in the scavenging effect of OH (Equations 4, 5).

$${\text{H}}_{2}{\text{O}}_{2}\;+\cdot \text{OH }\to {\text{ H}}_{2}{\text{O }+\cdot \text{HO}}_{2}$$ (4)

$${\cdot \text{HO}}_{2}\;+\cdot \text{OH}\to {\text{ H}}_{2}{\text{O }+\text{ O}}_{2}$$ (5)

The pH value is a significant factor in the TC-HCl degradation process. And the removal of TC-HCl by oxidation shows better performance in acidic condition than neutral and alkaline condition. It is probably because the surface of nZVI particles is prone to corrosion under acidic condition (Cao et al., 2018; Cao et al., 2019; Nie et al., 2020), and leading to a large number of Fe^2+^ release (Eq. 6). The increased Fe^2+^ reacts with H_2_O_2_ to generate more hydroxyl radicals, which promotes the oxidation of TC-HCl *via* Fenton-like reaction. As the increase of pH value, nZVI tended to form a passive oxide layer to block the reaction sites, and hence decreasing the degradation effect (Dong et al., 2018). In addition, H_2_O_2_ is easily decomposed into oxygen and water at alkaline conditions. As shown in Figure 8D, initial pH 5 has the highest TC-HCl removal efficiency. At pH = 5, the removal efficiency of 20 mg/L TC-HCl is 94.79% while 80 mg/L TC-HCl is 88.79%. However, when the pH value is too low, it will promote the release of hydrogen (Eq. 7), which produces air bubbles on the surface of catalyst. Moreover, nZVI can be able to dissolve rapidly in strong acidic condition, which limits the Fenton-like oxidation process as well as the production of H_2_O_2_ (Chen et al., 2020a).

$${\text{Fe}}^{\text{0}}{\;+\text{ O}}_{2\;}{+\;2\text{H}}^{+}\;\to {\text{ H}}_{2}{\text{O}}_{2}{\;+\text{ Fe}}^{2+}$$ (6)

$${\text{Fe}}^{\text{0}}{\;+\;2\text{H}}^{+}\;\to {\text{ Fe}}^{2+}{\;+\text{ H}}_{2}$$ (7)

### Kinetic Study

Generally, the pseudo-first-order kinetic model is extensively used to describe the Fenton-like process (Wang et al., 2021). The model can be expressed as follows:

$$ln\frac{C_{0}}{C_{t}}=kt$$ (8)

Where C_0_ is the initial concentration (mg/L), C_t_ is the concentration of TC-HCl at reaction time t (mg/L), k is the apparent rate constant (min^−1^), and t is the reaction time (min).

**FIGURE 9:**
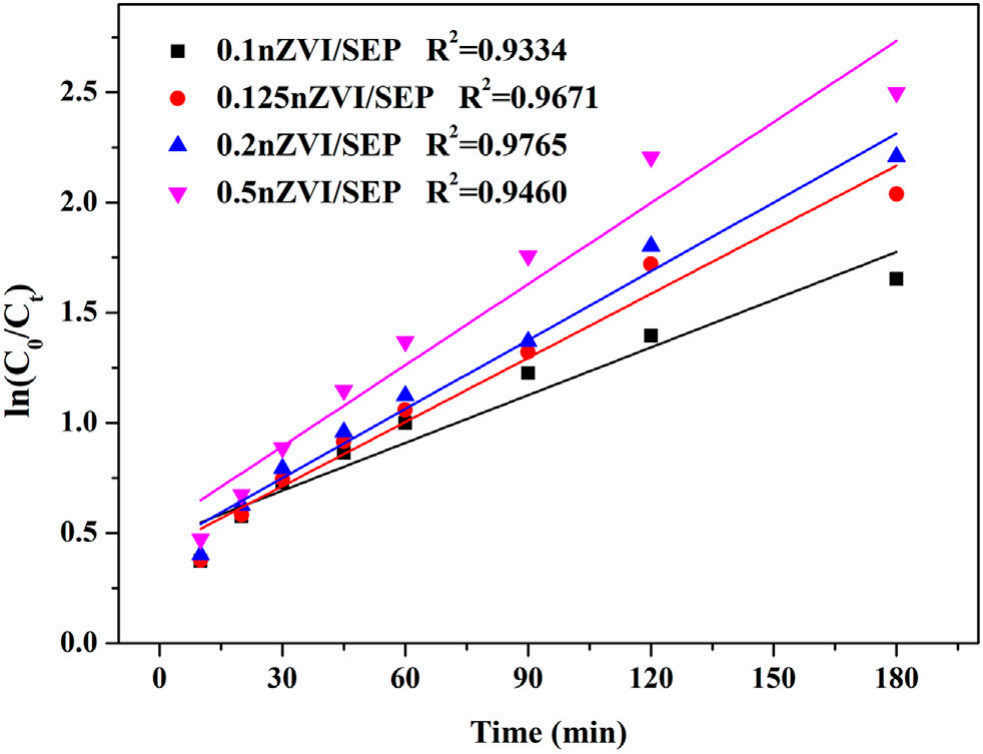
Pseudo-first-order kinetic curve of TC-HCl degradation. (Experimental conditions: TC-HCl concentration = 20 mg/L, catalyst dosage = 0.1 g/L, H_2_O_2_ concentration = 1 mM, pH = 7, room temperature).

Figure 9 shows the pseudo-first-order fitting curves of 0.1nZVI/SEP, 0.125 nZVI/SEP, 0.2 nZVI/SEP and 0.5 nZVI/SEP samples, and the corresponding reaction rate constant is 0.00722, 0.00969, 0.01042 and 0.01226 min^−1^, respectively. It reflects that the nZVI/SEP + H_2_O_2_ system is highly effective for TC-HCl degradation. And the reaction rate constant of TC-HCl degradation process is consistent with the previous reports (Zhang et al., 2018; Khodadadi et al., 2019; Xin et al., 2021). The 0.5nZVI/SEP composite exhibits highest catalytic efficiency, which indicates that the loading amount of nZVI is closely related to the catalytic performance of TC-HCl.

### Comparison of Tetracycline Removal Efficiency With Various Catalysts

The tetracycline removal efficiency of nZVI/SEP composite prepared in this work is compared to other related catalysts reported in literature and the result is listed in Table 2. It is clear that the nZVI/SEP composite displays excellent catalytic performance for tetracycline antibiotic under different conditions. Moreover, raw materials like green tea and sepiolite used in this work are cheap, easily available, safe and non-toxic. The synthesis process of nZVI/SEP composite is also simple and can be used in large-scale production. Hence, the synthesized nZVI/SEP composite can be employed as a suitable catalyst for the degradation of antibiotics in aqueous solution.

**TABLE 2:** Comparison of removal efficiency of various catalysts for tetracycline.

Catalyst	Catalyst dosage (g/L)	H_2_O_2_	TC (mg/L)	pH	Removal efficiency (%)	Reference
nZVI/SEP	1.0	1 mM	20	7	92.67	This work
Fe-MOFs	0.2	44 mM	10	5.0	83.3	Geng et al. (2021)
Fe^0^/CeO^2^	0.1	100 mM	100	5.8	91	Zhang et al. (2019)
Fe-biochar	1	5 mM	40	7.4	90.7	Bao et al. (2021)
Fe-loaded granular activated carbon	3.0	10 ml/L	10	2	87.01	Pan et al. (2019)
Fe-Mn binary oxide	0.4	1% wt	30	5	95	Chen et al. (2020b)
Mesoporous bimetallic Fe/Co	0.6	0.25 mol/L	30	7.0	86	Li et al. (2019)
Fe_3_O_4_ nanospheres	0.5	50 mM	25	7	82	Nie et al. (2020)
Fe-Co oxide nanosheet	0.3	20 mM	50	7	83.57	Nie et al. (2021)
Fe loaded graphitic carbon	0.02	1.0 mM	40	4.3	83	Wang et al. (2021)

### Passible Degradation Mechanism of TC-HCl by nZVI/SEP Composite

**FIGURE 10:**
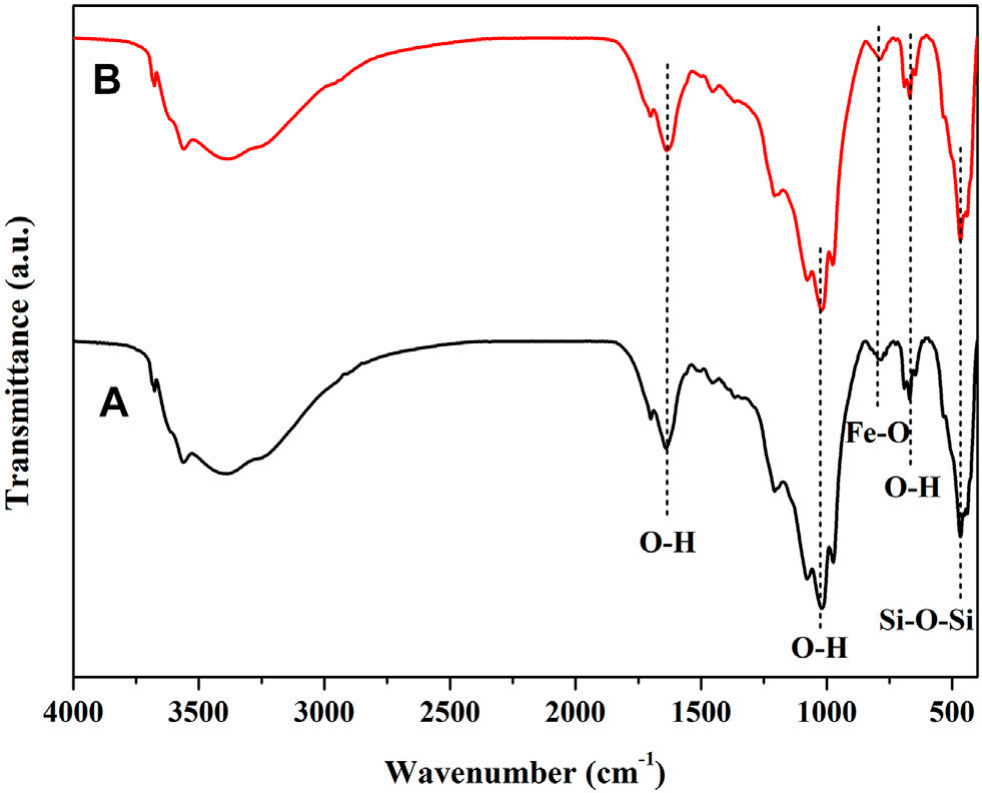
FTIR spectra of 0.5nZVI/SEP sample **(A)** before and **(B)** after the reaction with TC-HCl.

To investigate the interactions between the nZVI/SEP catalyst and TC-HCl, FTIR spectra of 0.5nZVI/SEP before and after the reaction were compared. As shown in Figure 10, there are no obvious characteristic peaks of TC-HCl in the spectrum after the reaction, which suggests that few TC-HCl is adsorbed to the catalytic surface. Consequently, only the degradation process in the reaction is discussed below.

**FIGURE 11:**
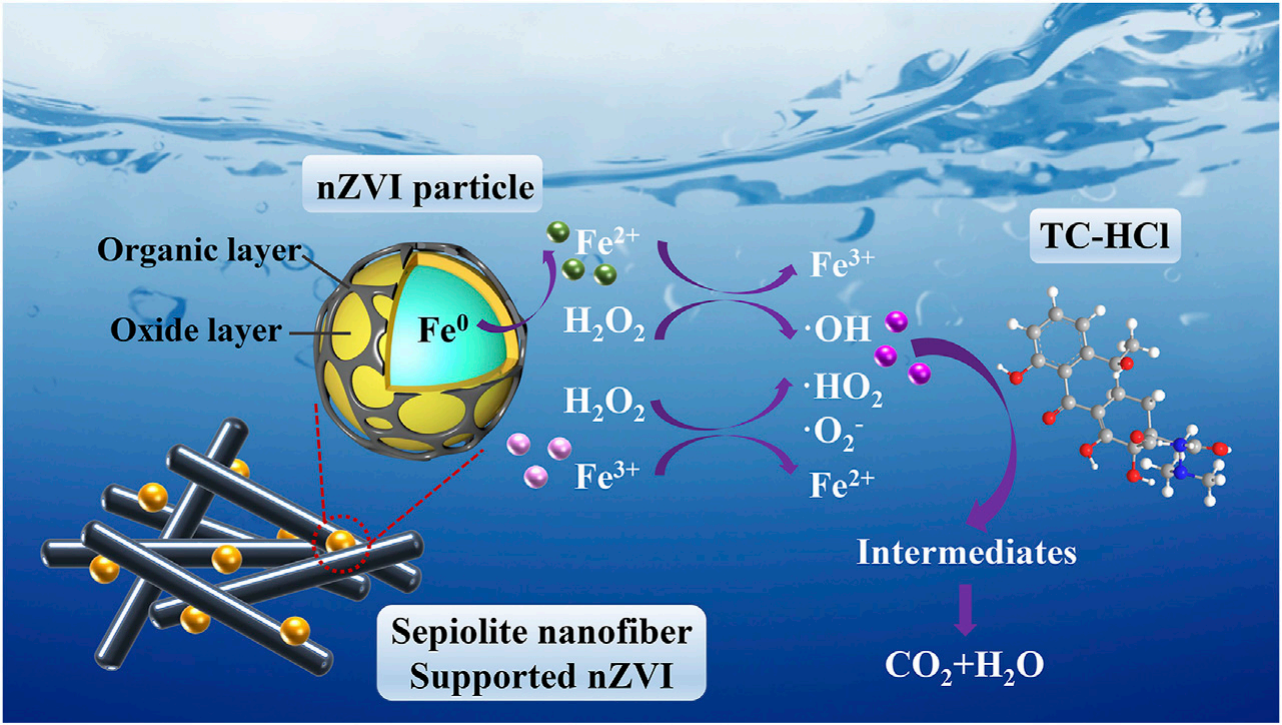
Removal mechanism for TC-HCl on nZVI/SEP composite in Fenton-like system.

Based on the experimental results and discussions above, a passible degradation mechanism for TC-HCl removal by nZVI/SEP composite in Fenton-like system is proposed and the diagrammatic sketch is depicted in Figure 11. The introduction of sepiolite can result in a better catalytic performance. On the one hand, sepiolite effectively inhibits the agglomeration of nZVI particles and reduces the size of nZVI particles, which can enhance the mobility and dispersion of nZVI particles and promote the direct contact between nZVI particles and contaminants in aqueous solution. On the other hand, the sepiolite supported nZVI composites possess the large specific surface area and porous structure, which is beneficial to conduct the contaminants to the reactive cites of the catalyst and then accelerating the degradation rate. Therefore, the NZVI/SEP composite is more efficient catalyst relative to either pure nZVI or sepiolite in removing TC-HCl antibiotic. In addition, there is a layer of capping agent (mainly amorphous carbon) on the surface of NZVI particles synthesized by green method, which can prevent the faster oxidation of Fe^0^ in practical application, and slow down the dissolution of NZVI particles because of the protection of surface capping. It leads to the slow release of active species to realize excellent degradation performance for antibiotic contaminants (Monga et al., 2020).

In the heterogeneous Fenton-like process, ·OH radical is the main ROS and plays a predominant role in TC-HCl degradation (Pan et al., 2019; Wang et al., 2021). Firstly, the addition of H_2_O_2_ accelerates the corrosion of Fe0, resulting in plenty of Fe^2+^ continuously produced (Eq. 2). Then the Fe^2+^ reacts with H_2_O_2_ and generates sufficient OH radicals (Eq. 3). And Fe^3+^ can be reduced to Fe^2+^ with the presence of Fe0 in the Fenton reaction (Eq. 9. In addition, the redox cycle of Fe^2+^/Fe^3+^ also promotes the generation of HO_2_/·O_2_^−^ radicals (Eqs 10, 11). During this process, because of the high specific surface area and nanofiber structure of sepiolite, it is beneficial to promote the direct contact between TC-HCl and catalyst, as well as provide more reaction sites for H_2_O_2_ activation. Finally, OH radicals on the surface of catalyst can efficiently degrade TC-HCl and the TC-HCl is degraded into some intermediates by OH and HO_2_/·O_2_^−^ radicals and eventually mineralized into CO_2_ and H_2_O (Luo et al., 2020).

$${2\text{Fe}}^{3+}{\;+\text{ Fe}}^{\text{0}}\;\to {\;3\text{Fe}}^{2+}$$ (9)

$${\text{Fe}}^{3+}{\;+\text{ H}}_{2}{\text{O}}_{2}\;\to {\text{Fe}}^{2+}{\;+\cdot \text{HO}}_{2}{\;+\;\text{H}}^{+}$$ (10)

$${\text{Fe}}^{3+}{\;+\cdot \text{HO}}_{2}\;\to {\text{ Fe}}^{2+}{\;+\text{ O}}_{2}{\;+\text{ H}}^{+}$$ (11)

## Conclusions

The zero valent iron particles were successfully loaded via green method onto sepiolite nanofiber at different nZVI/SEP ratios. It was observed that the spherical nZVI particles are well dispersed on the surface of sepiolite nanofibers with particle size of 20–60 nm. Moreover, the nZVI/SEP composite show larger specific surface area (101.35 m^2^/g) than pure nZVI (4.04 m^2^/g). Synthesized nZVI/SEP composites were applied for the degradation of TC-HCl antibiotic from aqueous solution. The nZVI/SEP composites exhibited higher removal efficiency of TC-HCl, which because the sepiolite inhibited the agglomeration of nZVI particles and improved the mobility and dispersion. Furthermore, sepiolite as support promoted the change of contact between high reactive sites and TC-HCl contaminant, thereby significantly increasing the catalytic activity. In order to explore the degradation mechanism of TC-HCl by nZVI/SEP composite. The effects of initial TC-HCl concentration, catalyst dosage, H_2_O_2_ concentration and pH value were also investigated. The efficient removal of TC-HCl was achieved in the catalyst/H_2_O_2_ system due to the combination of Fe0 reduction and Fenton oxidation processes. This work suggests that nZVI/SEP composite has great potential for remediation of antibiotic wastewater.

## Data Availability

The original contributions presented in the study are included in the article/Supplementary Material, further inquiries can be directed to the corresponding author.
